# Improving Health System Engagement in Patients With Substance Use Disorders: Audit of Care Plans in the National Drug Treatment Centre

**DOI:** 10.1192/bjo.2024.589

**Published:** 2024-08-01

**Authors:** Mary Keenan, Siobhan Rooney

**Affiliations:** 1 Central Mental Hospital, Dublin, Ireland; 2 National Drug Treatment Centre, Dublin, Ireland

## Abstract

**Aims:**

The National Drug Treatment Centre in Dublin is the longest established treatment service for drug users in Ireland. Led by Addiction Psychiatry teams, it provides specialist multidisciplinary input for patients with complex medical and psychiatric needs secondary to substance use. Many patients attending the centre engage poorly with other healthcare services in the community. The aim of this audit was to improve physical healthcare engagement in a caseload of 60 patients, through improving GP registration rates and possession of medical cards (providing access to free public health services).

**Methods:**

The comparison standard for the audit was the Irish Health Service Executive (HSE) Clinical Guidelines for Opiate Substitution Therapy: 2016. These stipulate that all drug users entering treatment and rehabilitation should have a care plan based on assessed need. Specific domains covered include: Drug and Alcohol use, Physical Health, Psychological Health and Social Functioning.

During the initial audit cycle, electronic care plans in a caseload of 60 patients were reviewed for information on their GP and medical card status.

Intervention: Following the initial cycle, results were presented and discussed at a multidisciplinary team meeting. A combined MDT effort was made to discuss medical card and GP status with patients during each interaction. Patients were referred to social work for support in application/renewal of medical cards and GP registration where required.

**Results:**

Results following re-audit:

The percentage of patients with GP registration clearly displayed on their care plan increased from 66% to 93%.

The percentage of patients with an up to date medical card increased from 12% to 45%.

The percentage of patients whose medical card status was unknown reduced from 72% to 44%.

The percentage of patients with their medical card number displayed on their care plan increased from 25% to 55%.

**Conclusion:**

There was a significant improvement in the number of patients with GP and medical card information documented clearly on their electronic care plan. This has assisted National Drug Treatment Centre staff in supporting patients' physical health needs more effectively, through close liaison with primary care providers and onwards referral to other services where required. It was noted that further efforts were required to build upon these results and reach 100% compliance. Recommendations and an action plan were developed to ensure ongoing improvement in standards.

